# ‘The way you talk, do I have a choice?’ Patient narratives of medication decision-making during hospitalization

**DOI:** 10.1080/17482631.2023.2250084

**Published:** 2023-08-24

**Authors:** Stine Eidhammer Rognan, Mie Jedig Jørgensen, Liv Mathiesen, Louise Christine Druedahl, Helene Berg Lie, Kajsa Bengtsson, Yvonne Andersson, Sofia Kälvemark Sporrong

**Affiliations:** a Department of Pharmaceutical Services, Oslo Hospital Pharmacy, Oslo, Norway; b Hospital Pharmacies Enterprise, Oslo, Norway; c Department of Pharmacy, University of Copenhagen, Copenhagen, Denmark; d Department of Pharmacy, University of Oslo, Norway; e Centre for Advanced Studies in Biomedical Innovation Law (CeBIL), Faculty of Law, University of Copenhagen, Copenhagen, Denmark; f Department of Pharmacy, Uppsala University, Uppsala, Sweden

**Keywords:** Hospital discharge, medication communication, patient perspective, shared decision making, Foucault

## Abstract

**Objective:**

Based on the principle of the autonomy of the patient, shared decision-making (SDM) is the ideal approach in clinical encounters. In SDM, patients and healthcare professionals (HCPs) share knowledge and power when faced with the task of making decisions. However, patients are often not involved in the decision-making process. In this study, we explore medication decision-making during hospitalization and how power in the specific patient—HCP relationship is articulated, as analysed by Foucauldian theory.

**Methods:**

A qualitative case study, comprising observations of patient-HCP encounters at an internal medicines ward at a university hospital in Norway, followed by semi-structured interviews. The narratives (*n* = 4 patients) were selected from a larger study (*n* = 15 patients). The rationale behind the choice of these patients was to include diverse and rich accounts. The four patients in their 40s–70s were included close to the day of presumed discharge.

**Results:**

The narratives provide an insight into the patients as persons, their perspectives, including what mattered to them during their hospitalization, especially in relation to medications. Overall, SDM was not observed in this study. Even though all the participants actively tried to keep their autonomous capacity and to resist the HCPs’ use of power, they were not able to change the established dynamics. Moreover, they were not allowed an equal voice to those of HCPs and thus not to escape the system’s objectification and subjectification of them.

**Conclusion:**

There is a need for HCPs to get more familiarized with SDM. The healthcare system and the individual HCP need to make more room for dialogue with the patients about their preferences. A part of this is also how health care systems are structured and scheduled, thus, it is important to empower patients and HCPs alike.

## Introduction

Patients and healthcare professionals (HCPs) have different types and extents of knowledge and power, complicating the decision-making process in clinical encounters (Gulbrandsen et al., 2016). In this study, the concept of shared decision-making (SDM) is used to investigate medication decision-making during hospitalization.

Elwyn et al. defined SDM as “an approach where clinicians and patients share the best available evidence when faced with the task of making decisions, and where patients are supported to consider options, to achieve informed preferences” (Elwyn et al., 2012) (page 1361). The concept draws on patient-centred care (PCC) (Elwyn et al., 2012), which is extracted from the holistic healthcare model and represents a shift from the conventional (biomedical) way of practicing medicine (Epstein, 2000). With the holistic healthcare model, patients are seen as experts on their own bodies, symptoms and situations, and therefore, they are increasingly invited to take an active role in their own care (Caron-Flinterman et al., 2005).

The traditional scientific discourses (such as the biomedical discourse) still dominate in clinical practice, and hence, patients are not involved in the decision-making process (Valles, 2020). One suggested reason is that HCPs have not been guided on how to accomplish PCC and SDM (Elwyn et al., 2012). To accomplish SDM, the power equilibrium needs to be dynamic and shifted from the HCPs towards the patients to obtain balance between the two parts (Elwyn et al., 2017).

A challenge to obtaining SDM is that many hospital-based HCPs make decisions before the patient is informed, and seemingly have limited focus on the emotional and relational dimensions necessary for SDM (Gulbrandsen et al., 2016). Furthermore, it is a challenge that some doctors claim to practice SDM despite studies showing that this is unaligned with patients’ experiences (Driever et al., 2020; Thevelin et al., 2022). Instead of SDM, the doctors tend to limit their discussion with patients to treatment options (Elwyn et al., 2012). To alleviate this, a simplified 3-step SDM model for clinical practice has been proposed (Elwyn et al., 2012), which also has informed numerous subsequent SDM models, as presented in the systematic review by Bomhof-Roordink et al (Bomhof-Roordink et al., 2019). The three steps are first: choice talk, which is the awareness that there is a choice, for example regarding treatment. This is initiated by either the patient or HCP. Second, option talk, which is when the patient is informed in detail about the treatment options. The third is decision talk, when the patient is supported to consider “what matters the most to them” after being informed (Elwyn et al., 2012). In addition to the simplified 3-step SDM model, other components include HCPs’ learning about the patient and the patient’s preferences before tailoring information. These are considered central components of SDM in almost any healthcare setting, however, a unified view on SDM is still lacking (Bomhof-Roordink et al., 2019). The view on what SDM entails may differ by healthcare setting, and often this is appropriate. However, the absence of a unified view limits training, implementation, policy, and research purposes (Bomhof-Roordink et al., 2019).

The difficulties of implementing SDM in clinical practice can be examined by applying concepts of the philosopher Michel Foucault. Foucault was concerned with knowledge, power, and the human subject (Heller, 1996; M, 2010; Ryan et al., 2004; Waring, 2007). Seen through a Foucauldian lens, the power of the field of medicine is positioned in the role of HCPs, while hospitalized patients also have to cope with illness and the existential challenges illness represents (i.e., uncertainty and vulnerability) and their implications for autonomy (Cheek & Rudge, 1994; Gulbrandsen et al., 2016; Juritzen & Heggen, 2006). Most patients trust the power of medicine and perceive HCPs as skilled (Gulbrandsen et al., 2016). Although some patients prefer that HCPs make the decisions, many want to take an active part. However, the person-in-power (for example, the doctor) might overlook how providing advice could mean making decisions on behalf of the patient, and further enhance the patient’s dependency and loss of autonomous capacity (Gulbrandsen et al., 2016).

Foucault described different types of power, including repressing power and normalizing power (Gutting & Oksala, 2019). The healthcare system is based on normalized power, which occurs when people automatically do what they must in order to comply with what society regards as normal (Cheek & Rudge, 1994; Gutting & Oksala, 2019; Heller, 1996). By contrast, repressive power is more visible and can be illustrated by people ordering around how others should act, for example, the police or the legal system (Heede, 2012; Leiden University, 2017). Repressive power in a medical setting could be changing patients’ medication without consulting them, not providing information about options and associated benefits/harms, not clarifying congruence between decisions and personal values (Gulbrandsen et al., 2016; Gutting & Oksala, 2019; Juritzen & Heggen, 2006). The patients’ resistance towards the system may arise when the assumed powerless patients try to rebuild their autonomous capacity by being more active, speaking up for themselves, and making demands (Gulbrandsen et al., 2016; Juritzen & Heggen, 2006).

The focus on articulating power in the specific patient-HCP relationship from the patient perspective has been limited (Tomlinson, 2018). Sociological analysis of the medical landscape has been dominated by studies on the practices and discourses of HCPs, narrated from their perspectives (Agner & Braun, 2018; Tobiano et al., 2019; Tomlinson, 2018). From the patients’ perspective, by analysing narrative case descriptions from a Norwegian hospital setting, we aim to understand how patients are involved in SDM and how power operates in the relations between the patient and the HCPs.

## Materials and methods

### Research design

In a previous study at an internal medicines ward (Lea et al., 2020), the clinical pharmacists perceived the discharge process as complex and with many actors. To understand the hospital discharge process better, a qualitative study (Bengtsson et al., 2022; Rognan et al., 2021b). was designed to be performed at the same ward. This study especially focussed on the medication communication and the patient perspective.

The data collection consisted primarily of non-participant observations at an internal medicines ward in Norway, and semi-structured interviews with patients after discharge. Data was collected from September 2019 to December 2019. For more details see articles by Rognan et al (Bengtsson et al., 2022; Rognan et al., 2021b, 2021a). In the current study, the purpose was to identify significant patient experienced events related to day-to-day interactions and SDM (Beal, 2013; Moen, 2006; Polkinghorne, 1995). For this purpose we included cases that we considered to be rich on day-to-day interactions and SDM.

## The researchers’ backgrounds

Multiple authors with different backgrounds (education and experience) contributed to building and analysing each narrative to obtain a nuanced analysis. However, all are female, all have a Northern-European background, and all except YA and SKS are trained as HCPs (pharmacists and clinical pharmacist). The authors are experienced from early to advanced training in qualitative research and its processes. Much of the work was performed by pharmacy students to facilitate that more of the naïve questions were asked to the material from the observations and to the patients in the interviews (Malterud, 2011). Team discussions was an important routine during the data collection period.

## Data collection

Observations were conducted by HBL, KB, and SER. They observed all encounters between patients and HCPs potentially including medication communication, Monday to Friday (and occasionally in weekends) from 8:00 to 15:30, covering the period when most hospital activities normally take place. Summarized characteristics of the observed HCPs on the day of discharge are presented in a previous publication. Although the specific roles of the HCPs were collected, the broad term is generally used as it, from a patient perspective, is an encounter with the medical team as a whole. The observed participants knew the observers’ background (pharmacy student or clinical pharmacist at the internal medicines ward), and each observer was identifiable, but without any role in the social setting (Richards & Emslie, 2000). The observers wore a yellow t-shirt with the word “observer” across the front (Brannick & Coghlan, 2007; Richards & Emslie, 2000). Data from the observations were documented in an observation form, but only audio-recorded if occurring in a patient single room and if both the respective patient and HCPs had consented. The use of reflexive field notes added to the descriptive nature of the observations and made the observers’ contributions more explicit (Maharaj, 2016). Reflexive field notes consisted of impressions, subsequent thoughts that moved the observer, and how the presence of the observer potentially influenced the participants.

Semi-structured interviews were conducted with patients 1–2 weeks after discharge, focusing on the patients’ experiences of medication communication at the hospital (Simonÿ et al., 2018). The design was also chosen to limit or prevent imposing the observers’ preconceived notions on to the data and allowed the patients to express freely (Aase et al., 2013; Mach et al., 2005; Miller et al., 2018). The interview guide can be found in a previous publication. The broad topics of the interview guide were based on the overall research aim and literature on medication communication in hospital. The open-ended questions concerned the patient’s experiences and expectations: regarding general information at the hospital, being observed, the hospital discharge, medication information, beliefs about medications and the period after hospital discharge. During the development of the interview guide, it was tested within the research group. The guide was adapted to each patient depending on his/her experiences at the hospital (Rognan et al., 2021b). The interviews were audio-recorded if the patient had provided an additional consent. All data were transcribed verbatim.

## Sampling strategy

The sample size was not predetermined, but depended on reaching sufficient information power (Malterud et al., 2016). To ensure data heterogeneity in the larger project, the participants were hospitalized patients that were selected on socio-demographics (such as gender, age, education, and ethnicity), diagnoses, and assumed length of hospitalization. Inclusion criteria were: ≥18 years of age, home dwelling, taking responsibility for their medication administration prior to hospital admission, and expected discharge to home or short-term nursing home. Pre-terminal or cognitively impaired patients were ineligible. Eligible patients were continuously compared with data from previously enrolled patients to ensure that different patient characteristics were covered, for example various age categories (Malterud et al., 2016). After fifteen included patients, we agreed in a consensus discussion with five analysers present, that we had included a heterogeneous sample in terms of diversity in demographics, medical history and medical treatment, and that no significantly new insights could be drawn from further data collection. From the overall sample, four patients were selected, see below.

## Selective sampling strategy

In this sub-study, the narratives (*n* = 4 patients) were selected from the larger study (*n* = 15 patients). The selection of four cases to constitute the narratives were justified by the rich data material present in these cases, and an opportunity to present in detail, from the patient perspective experiences at hospital discharge. The four selected narratives also represented variability in gender, ethnicity, age, cause of admission and education. The selection aimed to 1) get a heterogeneous sample of a variety of data expressed across age, gender, and experiences such as medication communication, length of stay, total duration of observed HCP encounters, single or multi-bed room, and duration of interview (Connelly & Clandinin, 1990), and 2) include narratives with rich descriptions of context and web of social relationships, both during hospitalization and after discharge (Moen, 2006). The narratives should provide insight into the voice of each patient, i.e., individual patient perspectives and actions related to the phenomenon of interest (Connelly & Clandinin, 1990).

Audio-recorded encounters for 2–8 days were available for the patients in the sub-study. All encounters were audio-recorded, except for the first 22 days for one patient that initially stayed in a multi-bed room. This did not alter the analysis as we see it, because the data material in this case was rich compared to other patients that were observed fewer days.

## Data analysis

The approach to the analysis was narrative, hence emphasis was not placed on thematic categorization of data, identification of similar patterns or to achieve saturation. MJ and SER analysed the data using the following steps:

1. What is said and/or observed. The focus was on what patients met during a hospitalization and how they experienced the medication communication. For each case, the transcripts (observations and interviews) were read chronologically to get an in-depth understanding of the text as a whole and to identify essential characteristics and patterns (naïve reading) (Beal, 2013; Simonÿ et al., 2018). Notes were made on immediate impressions, and events that moved an analyser were highlighted (Simonÿ et al., 2018; Smith & Smith, 2016).
2. What the text concerns. The text was read again as a whole, making highlights and notes in light of the interpreter’s pre-understanding, including SDM, and Foucault’s works. The narratives evolved inter-subjectively, with the patient’s voice, coloured by the researchers’ reporting and analysing (Connelly & Clandinin, 1990; Smith & Smith, 2016; Wang & Geale, 2015). This plurivocalness in overlapping narrative constructions resulted in the final format of the narratives (Connelly & Clandinin, 1990).
3. Structuring the narrative scene and plot. The three-dimensional space narrative structure approach was used to describe the characteristics of each patient’s experiences in a personal and social context (Wang & Geale, 2015). This implies that the dimensions of interaction, continuity, and situation were in focus. Where applicable, experiences were grouped according to past (pre-admission), present (during hospitalization), and post discharge (Connelly & Clandinin, 1990; Ollerenshaw & Creswell, 2002).
4. The four patient narratives were created from the excerpts that illustrated each case (Connelly & Clandinin, 1990).

LM, LCD and SKS provided feedback on the narratives for validation. Furthermore, LM, LCD, HBL, KB, YA and SKS assessed if the content of the narratives seemed reasonable, for example whether the balance in the patient narratives had been properly presented without too much fragmentation, in view of possible alternative stories (Connelly & Clandinin, 1990; Wang & Geale, 2015). The narratives were circulated among the research group to consciously discuss the selections made and to fine-tune the final format. Hence, the final version of the narratives presented here is the result of several rounds of discussions among the researchers.

Consistent with narrative inquiry (Connelly & Clandinin, 1990) we present the results in a storied form. Please note that to maintain anonymity, all names have been changed and that quotes are from the interview unless marked with (obs) for observation.

## Ethical considerations

Written informed consent from all patients and HCPs (nurses, nursing assistants, doctors, medical and nurse students, physiotherapists, and clinical pharmacists) were obtained before inclusion into the study, by the observers. The Regional Ethics Committee assessed the study and found no ethical approval necessary. The study was approved by the Privacy Ombudsman and the Hospital Investigational Review Board on 8 March 2019, reference number 2019/6465.

## Results and discussion

The findings show how four patients in their 40s, 50s, 60s, and 70s, respectively, experienced parts of their journey during their hospitalization towards the hospital discharge. The data material consisted of 471 pages of transcripts obtained from about 45 hours of observed patient-HCP encounters. For each patient, 0–2 interviews were performed. The average length of the total number of interviews was 69 minutes (range 24–87). More details are presented in Table I.

**Table I.** Participants, duration of observation, duration and number of interviews.

Participant^a^, gender	Age	Observed days^b^	Total duration of observations (min)	Observed discharge	Number of semi-structured interviews	Length of interviews (min)	Total number pages of transcripts (observation + interview)
Elena, ♀	40s	2	270	1	-	-^d^	37 (37 + 0)
Simon, ♂	50s	4	110	1	1	87	82 (23 + 59)
Karen, ♀	60s	22	2167	-^c^	2^c^	24+86	215 (171 + 44)
Peter, ♂	70s	3	172	1	1	78	42 (26 + 16)

Note: ^a^The participants’ names are pseudonyms.

^b^One third to half of the patients’ hospital stay was observed.

^c^Karen was not observed at hospital discharge due to transfer to another ward before discharge and lack of consent from HCPs at the new ward. Due to transfer to another hospital ward, she was interviewed twice.

^d^Elena was not reachable after discharge, hence the interview was not carried out.

## Elena’s narrative

### Background

Elena is a woman in her 40s who has a higher education and lives with her family. Her parents were both of a non-European background. Elena had a therapy-resistant migraine, for which she was previously treated with triptans, and pre-admission she was treated with high dosage prednisolone (according to Elena to treat triptan withdrawal symptoms). In the months before the hospital admittance, she experienced thirst, mouth dryness, increasing nausea, headache, and weight loss. For this, Elena’s GP prescribed metformin due to her high blood sugar level, but the treatment was terminated due to troublesome side effects.

### Hospitalisation

During the hospitalization, Elena was formally diagnosed with type-II diabetes, and she received treatment with high dosage prednisolone.

Elena’s encounters with HCPs were characterized by being student-driven, with nursing and medical students practicing mostly independently of their supervisors. Elena discovered that during her hospitalization the HCPs had provided her with a too high dosage of prednisolone.

Elena: “I got 15 [mg] today, 10 [mg] yesterday. So I got a little confused because I thought I was going to taper down the dosage”. (obs)

Elena: No, my neurologist suggested it [prednisolone], it should be like a wash-out cure. Ehm, because I had used so much triptanmedication (against migraine) and that it was still in my body. And I started with it [prednisolone] in April and (I) got well, so we went back to it [prednisolone]. (obs)

During her hospitalization, Elena showed a wish to keep some sense of control regarding the medical treatment options, about which she had opinions. Furthermore, she asked questions to the HCPs to make them elaborate on the reasons behind the decisions they made about her treatment.

Elena and the nursing student with who she had most contact seemed to have an especially good and friendship-like connection, for example, observed from them laughing together. One time when Elena had a nosebleed accompanied by tingling pain in her face, the nursing student answered on Elena’s behalf during the doctor’s examination, when Elena seemed to be too sick to express herself. In another observation, the nursing student verbally supported Elena when the doctors left the room abruptly without saying goodbye, even though Elena clearly indicated a wish for more help. Moreover, the nursing student taught Elena how to self-manage her insulin pens [for diabetes], but also provided her with some incorrect information.

### Discharge

During the discharge conversation, Elena expressed that she did not feel ready for discharge.

Elena: ‘Oi, 14.3.

Nurse: Yes, the more often you measure (the blood sugar) after you have eaten, the higher it gets.

Elena: Yes, yes. But I feel that I am sicker.

Medical student: With the measuring?

Elena: No, I feel that I am shaking inside. My entire body is shaking.

Medical student: Yes, okay. You might just be a little anxious.

Nurse: Then she had a question, so I just…

Medical student (interrupts): Can you print out the discharge summary?’ (obs)

It was observed that Elena explained to the doctors about the too high dosage of prednisolone in her current treatment more than once. However, during the discharge conversation, it became apparent that the HCPs saw this as “new information” that they had not considered in the earlier encounters. During the discharge conversation, in which a medical student, the nursing student, and the nursing supervisor participated, Elena expressed concerns about being unable to self-manage the dosages of her insulin. The medical student interrupted Elena while she was talking and did not acknowledge Elena’s concerns, and then actively refused her request for extra guidance.

Elena: *‘Yes, I just thought, now I know I can’t stay any longer. But can we wait until the next time I have measured my blood sugar?*

Nurse: *Yes, if you collect the equipment at the pharmacy, we can look at it together around 16 o’clock.*

Medical student: *But will that make any difference? You have to adjust the dose yourself eventually anyway’. (obs)*

After the discharge conversation, Elena was still uncertain about how to manage her insulin. The written information about insulin in the discharge summary was: “adjust as needed, in accordance with the sliding scale”. The nurses though “allowed” Elena to stay some hours longer at the hospital, to have her blood glucose measured one more time under their supervision.

## Interpretation and discussion of Elena’s story

- **Worried, seeking relations but not getting involved**

### SDM

The HCPs were more focused on Elena’s migraines (i.e., on prednisolone cures, etc.) rather than on empowering Elena to self-manage her medications. Shared decision-making was not observed between Elena and the medical student, who appeared to be the primary consulting doctor. Elena sought an emotional/relational approach regarding her treatment options and wanted to have a dialogue with the HCPs, but she was not involved and hence her wishes remained unmet. Elena was not informed about any of the changes made to her medical treatment during the hospitalization (option talk) and thereby could not make a decision (decision talk) based on what mattered to her. It is unclear how the friendliness between the nursing student and Elena should be interpreted, but it was evident that they had a more emotional dimension to their conversations.

### Foucault

Elena’s journey shows that there is a seemingly large difference in the position of power between doctors and Elena, but less so between the nursing student and Elena. In addition, Elena showed resistance to the current power relations by not “just saying yes” to all treatment decisions, but questioning the HCPs and asking to be involved.

## Simon’s narrative

### Background

Simon is a highly educated Northern European man in his 50s. He did not understand Norwegian but mastered English above average. Simon lived with his wife in his native country, when he was not on one of his frequent business trips to Norway. Before the admission, Simon had been using five medications on a regular basis (against hypertension, chronic kidney disease, and asthma) and one on demand (against asthma).

### Hospitalisation

Simon was admitted due to multiple pulmonary embolisms. At the beginning of the hospitalization, Simon had felt excluded from the decision-making. He told this during the interview after discharge. However, he reflected on not having been fully conscious and not able to participate in decisions at that time. He suspected that if he had received information about medications early in the hospital stay, he would have been too sick to understand it and take an active part in the decisions. He became more comfortable being a part of the decisions regarding his medication and health as he recovered.

When we started observing Simon, he was very talkative and sometimes the conversations were dominated by Simon who could go from one subject to another. He tended to interrupt other people while they were talking, including HCPs, but they often let Simon continue to talk. Simon wanted things to happen fast, preferably with a schedule.

In the interview, Simon expressed a pre-understanding of HCPs that was strengthened by experiences during his hospitalization:

Simon: I think that one of the doctors that they brought in did not speak anywhere near as good English, and, eh, I do not want to be rude, but in my home country consultants have a reputation of not listening and knows everything. So I do not know with her, I did not feel that she listened to what I said, and did not answer my questions and whether that was, she had a prepared speech in English that she wanted to deliver and did not understand me, or whether it was consultant behaviour.

Simon also told that a disadvantage of this hospital was that there was frequent rotation of HCPs because he appreciated seeing a “*known, friendly face*”. In addition, Simon expressed that some of the HCPs were not very talkative due to language barriers. For example, one nurse walked out of the room when she realized Simon did not speak Norwegian. The observer also noted that some HCPs asked for help to translate words into English. Furthermore, Simon felt that the interest from the HCPs in helping him dropped, when they were handed his EU health card because he was not a Norwegian citizen. The HCPs wanted to do several examinations that were not financially covered in the current agreement of care between Norway and the EU.

In the interview, Simon told that he had felt more in charge at the end of the hospitalization. He also told that it was important to him to understand “*the bigger picture*”, so he “*pushed*” the doctors to give him information. In addition, he used what he called “*google—with sense*”, i.e., looking up conditions and medications to see how the medications worked and if they would interact with each other.

### Discharge

Simon thought that his discharge probably had been delayed due to language barriers, paperwork, and miscommunication. This was exemplified by his experience on the day of discharge where he waited for his doctor to return as promised. The doctor said that he would return to confirm that everything was ready so that Simon could leave, but no one came; after about four hours Simon asked a nurse if he could leave, which he could.

Simon: “On the day of discharge, the information was a bit lacking, it was just ‘yes you can go’”.

In the interview, Simon told that he still had unanswered questions. The discharge summary had been translated into English, but still had some Norwegian words and medical terms in the medication list. In addition, the explanation for why one of his medications was discontinued was lacking.

## Interpretation and discussion of Simon’s story

- **Fighting for involvement, asking for continuity**

### SDM

Simon took part in his own treatment, however, not to the extent he wanted. He did not feel fully informed about his treatment options because the information was not detailed enough for him. Simon reflected on this and he was aware that he was too sick at the time of hospital admission to take part in the decision-making. Nevertheless, he also knew that when he was able to, he wanted to take an active part in the decisions regarding his treatment. Simon expressed understanding of how power works in the healthcare system. This may be due to his position in society since he was experienced in working with management.

### Foucault

Simon reflects on the HCPs’ approaches, the relations between him and the HCPs and among themselves. He was also clear about the potentials for improvement, such as seeing a known friendly face, better communication at discharge as crucial for empowerment, for example, to get all questions answered. Thus, Simon’s awareness of power relations and knowledge as power made him gain a higher position of power as a patient. Simon appeared reluctant towards the biomedical discourse.

## Karen’s narrative

### Background

Karen is a woman in her 60s, retired, living with her husband. She cares a lot about her family and the people around her. She is (almost) always positive and often smiles and laughs. She wants to live her life and to stay independent. Karen was admitted to the hospital due to heart failure. She also had multiple comorbid medical conditions, including type-II diabetes with complications.

### Hospitalisation

The first three weeks of her hospitalization, Karen shared a room with three other patients, her privacy being guarded by a curtain. Karen saw these three other beds in the room being occupied by different patients who came and went. Karen did not experience the same rapid recovery. Later on, Karen was moved to a single room. Overall, as the days and weeks passed by, Karen generally had the ability to keep her spirits up and to enjoy small things like apricot jam, the radio, and her family visiting her. For the last weeks of her hospital stay, she was transferred to another ward for a second opinion on her heart condition.

When Karen was admitted, one HCP asked Karen “*what matters the most to you*?” (obs). Karen responded by highlighting the value of a few HCPs to relate to during her hospitalization. During her first five weeks in the hospital, however, Karen was in contact with more than fifty different HCPs including nurses, nursing assistants, doctors, medical and nurse students, physiotherapists, and pharmacists.

Karen made it clear in the HCP encounters what mattered to her. Firstly, she valued being talked to in first person, and not in third person. Moreover, she wanted to remain in some control over her medication, and she wanted to be self-reliant and able to carry out normal activities after discharge. Karen asked questions and provided the HCPs with feedback. She also challenged the HCPs that had made decisions on her behalf:

Karen: What are you talking about now? [Karen asking HCPs who were talking to each other]

Doctor: We put you on a medication that is more potent, you have good absorption of the tablet, but we want to give (it) intravenously, that is better. (obs) What are you talking about now? [Karen asking HCPs who were talking to each other] Doctor: We put you on a medication that is more potent, you have good absorption of the tablet, but we want to give (it) intravenously, that is better What are you talking about now? [Karen asking HCPs who were talking to each other]

Doctor: We put you on a medication that is more potent, you have good absorption of the tablet, but we want to give (it) intravenously, that is better

Karen: ‘Are you not supposed to go through the medications?

Nurse: Oh, yes, thought you had control since you did not ask.

Karen:Everyone usually do it. Nurse: Blood-thinning, painkillers … Karen: Painkillers?

During the first days of her hospitalization, Karen was repeatedly informed that she could go home “any day now”. However, her condition worsened and she also experienced anxiety. She asked a doctor to get psychological help, and the doctor replied that she could talk to the hospital pastor. Afterward, she expressed that this had not helped her. When Karen experienced additional symptoms and was conveyed bad news about illness progression, her interest in remaining in control of her medications declined. For several days, Karen was afraid of never being able to leave the hospital and that she would die there. At the same time, which appeared to make her feel worse, she suspected that the HCPs withheld information on her condition from her.

Karen: “What is happening, am I dying?” (obs)

Karen: ‘The bad thoughts continued again. I thought that the reason behind their decision of giving me a single room was so that I should die alone and that it was the end. (obs)

Doctor: That was bad communication. Bad communication from us. That was absolutely not the thought.’ (obs)

Several days passed before the doctor, whom Karen saw most, managed to clarify that the reason for moving her to the single room was not what Karen thought, i.e., that she was going to die. The doctor explained to Karen that she had tried to put herself in Karen’s position, with other patients coming and going as disturbing environmental factors, and thus decided to move Karen to a single room. The intention seemed considerate, but the HCPs failed to communicate the reason which caused Karen to misinterpret the situation. A few days after this, Karen was moved to another hospital ward to get a second opinion on her heart condition by other specialists. Hence the daily observations terminated.

### Discharge

Following the stay at the other ward and ten weeks after being admitted to the hospital, Karen was discharged to her home. The discharge was not observed. In the interview (after discharge), Karen reflected on how she had not felt ready for discharge. Her request to stay for one more day to be able to ask questions had been dismissed.

Karen: ‘To them [the doctors], they [patients placed in the corridor] were next in line’

Karen’s discharge summary was not written in a lay language, and Karen had difficulties understanding it. The main text stated that the rifampicin (against infection) should be continued for two more weeks, however, rifampicin was not on the medication list in the discharge summary. In the interview, Karen told that she did not use the medicine and also that she lacked knowledge about it.

## Interpretation and discussion of Karen’s story

- **Seeking to be seen as a whole being, wanting options**

### SDM

The hospital routines seemed more important to HCPs than Karen’s lifeworld and specific needs, which were both central to her and communicated by her. For example, she requested option talk and protested when HCPs neither involved her in the choice nor in decision talk. The HCPs often made changes in the treatment without consulting Karen and talked above Karen’s head, hereby objectifying Karen. For example, Karen was placed in a single room to be less disturbed, without being involved in the decision. As a patient, she perceived that her experiences and needs in the situation were not taken into account. Relations mattered a lot to her and the lack of psychological support made her more vulnerable. The severity of her illness and the resulting existential journey may have reduced her autonomous capacity and thus her ability to self-manage her life.

### Foucault

Taking the perspective of Foucault, Karen was placed in a single room to be less disturbed without being involved in the decision, which made her a “docile subject” (43). Due to the severity of her illness, she experienced an existential journey affecting her capacity. A period of powerlessness led to a marked power shift from her to the HCPs as Karen lost interest in her treatment. Furthermore, the HCPs often made changes in the treatment without consulting Karen and talked above Karen’s head, hereby objectifying Karen. Karen tried to resist HCPs’ use of power to keep her autonomous capacity, which occasionally seemed to succeed.

## Peter’s narrative

### Background

Peter is a man in his 70s, retired, living with his wife. They were self-reliant besides receiving some practical assistance with housekeeping, and Peter expressed a desire to continue like this. He was an outgoing person who seemed to enjoy making other people laugh. Due to heart failure, gouty arthritis, and comorbidities, he had extensive experience with being a patient. Peter was treated at different health clinics and was experienced in receiving different kinds of information regarding his treatments. Lisinopril against heart failure had been discontinued (for some time) by his GP as Peter experienced severe side effects. Before the admission, Peter used prednisolone against his gouty arthritis that was painful and a torment to him.

### Hospitalisation

He was admitted to the hospital due to severe pain in his knees, ankles, and from under his feet. He had increased pain and swelling in the joints, despite undergoing treatment during this hospital stay, Peter’s treatment with lisinopril (and prednisolone) was being both prescribed and discontinued several times.

Peter was eager to seek medication information from HCPs, such as name, indication, and dose, during the entire hospitalization. At the admission, which was at the end of the week, the HCPs discontinued prednisolone and prescribed methylprednisolone, which was intended to be more favourable considering Peter’s heart failure. HCPs did not tell Peter that prednisolone had been discontinued. In the interview, Peter could not recall having this explained to him.

During the weekend immediately after his admission, Peter mostly met with nurses, as the doctors were not staffed to go on ward rounds during weekends. Saturday morning, Peter found out that the HCPs were about to administer lisinopril. Peter showed his frustration as he explained to the nurse about the agreement he had with his GP.

Peter: Should I take Lisinopril now? thought it was taken out because of the heart in consultation with the physician? had made an agreement with my general practitioner, reduced down from 2 to 1 tablet and then removed. I became so dizzy from it. Should I take Lisinopril now? thought it was taken out because of the heart in consultation with the physician? had made an agreement with my general practitioner, reduced down from 2 to 1 tablet and then removed. I became so dizzy from it.

Nurse: “I will talk with the physician who visits you today, then we will see what we shall do. I have to find out how this looks, then I will take it out.”

After the nurse had forwarded the information to the doctor, the nurse could tell Peter that he did not have to take the medication after all.

### Discharge

On Monday morning, the day of discharge, Peter met a doctor who talked him into using lisinopril again.

Peter: ‘Lisiniopril… Must I take it no matter what or? Is it the heart or?

Doctor: Yes, it is so that you have a history of a not inconsiderable degree of heart failure.

Peter: Yes, yes, yes.

Doctor: And Lisinopril would be very favourable to use. If we have to take away something that lowers the blood pressure it should rather not be Lisinopril. That is… very favourable for the future of the heart, one may say.

Peter: Is there something else which can… with blood pressure and dizziness?’

At the discharge conversation, the doctor sat down with Peter to explain the changes in the medication list in the discharge summary, including that “*the changes are marked in bold*” (obs). During the conversation, Peter asked for cooperation between the hospital and his GP so that they had the same details about his medication and the treatment decisions made. He was told by the doctor that this was not possible due to different software systems.

According to Peter, the HCPs sent him home too early due to a lack of resources. Peter experienced insufficient communication related to his medical treatment despite his initiatives to improve the cooperation between HCPs at the hospital and his GP and his requests for detailed information. In addition to the back and forth decisions with lisinopril, communication about the newly started methylprednisolone cure, inaccurately described as “prednisolone” or “cortisone” by doctors and nurses during the hospitalization and on the day of discharge, was confusing After discharge, both Peter and his wife (who was present in the room where the interview took place) expressed that unclear information about medications made them uncertain as to when the responsibility for the administration of medications was transferred back to them after hospital discharge. Peter still used prednisolone, which could be due to unclear communication at the hospital. Peter had been to his GP after the discharge, who solved the lisinopril-related problem with dizziness and ankle stiffness by altering the dose.

Peter: ‘We [the GP and I] decided that I could manage with half a dose’

## Interpretation and discussion on Peter’s story

- **Frustration, seeking cooperation between HCPs**

### SDM

Peter was not fully informed or involved in the decisions about his medication and the changes that had been made, which could have led to inappropriate self-medication. Even though the HCPs seemed to listen to Peter, changes were made without an HCP talking with him or informing him. This excluded an appropriate SDM. Peter also asked for more information sharing between the different health institutions involved in his care but was told that the different institutions do not share information. This illustrates a hospital discharge process where the patients are held accountable for sharing all the information with other HCPs which they might not be able to (Flink et al., 2012), especially in Peter’s case because he was not informed properly.

### Foucault

Peter was talking about the possible structural power behind the decision of his discharge, i.e., that it was due to lack of resources. A difference can also be seen in the power relations between Peter and HCPs at the hospital versus Peter and the GP. At the hospital, the HCPs did not listen to Peter regarding lisinopril, which his GP did. If the hospital HCPs had used SDM, they might have understood why Peter did not want to use lisinopril (Elwyn et al., 2012). Personalized and balanced communication about the risks and benefits of medical treatment during the hospitalization was lacking, which is unfortunate as it put Peter in an inferior knowledge and power position in relation to the HCPs.

## General discussion

The results provide an insight into the patients as persons, their perspectives, including what mattered to them during their hospitalization, especially in relation to medications. This included self-management, empowerment, and being involved in decision-making and decisions.

Overall, SDM was not observed in this study even though all four patients actively sought more information, asked questions and talked about their personal preferences and circumstances, factors known to facilitate SDM (Joseph-Williams et al., 2014). Although the patients challenged the HCPs by asking why they were not informed about changes and decisions taken on their behalf, they experienced less optimal communication and their needs and requests not being heard. Examples of needs not being heard include Simon’s requirement for medication information and Karen’s need for involvement in the decision-making process. HCPs not listening to patients increase the potential for medication harm that could have been prevented, for example intentional or unintentional non-adherence when the patients self-manage their medications post discharge. Without good and inclusive communication, both parties equally talking and listening to each other, SDM cannot take place from a patient perspective, as the HCP is in power (being on their “home turf” in relation to patients).

It has been suggested that patients prefer different healthcare models, and that it might be necessary to identify which healthcare model each individual patient prefers (Swenson et al., 2004). There is also a question if HCPs share the same view and/or accept the needs expressed by the individual patient. It has been shown that when a patient and doctor share the same view on SDM there tends to be a more positive patient outcome, for example a higher satisfaction among patients (Jahng et al., 2005).

It is, from a patient perspective, hard to differ between (specific models of) SDM and better communication. Some decisions are “clear”, and can be observed by comprehensive “bullet point” SDM models. But many are not and are taking place during the course of hospital stay, which for some patients in this study were rather long. Remarkably, good and inclusive communication and focus on the person facing the decision is very often absent in SDM-studies and because of this there has been calls to extend the conceptualization of SDM (Bomhof-Roordink et al., 2019; Rake et al., 2022).

The HCPs´ perspectives can indirectly be seen from the participants’ narratives. HCPs’ barriers to accomplishing SDM seemed to be complex, comprising factors such as discourses, organizational staffing pressure, handover between clinical shifts, hospital policies on discharge, pressure on individual HCPs, patient and HCP knowledge, experience, and personalities. Changing people and established practices is challenging. Previous studies have shown that even if doctors preferred or reported high levels of SDM, they most often practiced paternalistic decision making (Driever et al., 2020; Thevelin et al., 2022).

Circumventing SDM can make it easier for the HCPs to avoid sensitive topics, such as death, and to make alterations and decisions first and tell the patient about these afterwards. Another aspect is whether HCPs are able to practice SDM, exemplified by Karen’s narrative, where the large number of HCPs involved may have impaired the strength of patient-HCP relations, and hence represented a barrier to practice SDM. From Elena’s narrative, HCP students’ uncertainty and lack of knowledge could explain the lack of SDM. HCPs´ expressions of uncertainty have been found to reduce patients’ trust. The HCP students may have been inexperienced and uneasy or even afraid of “exposing” knowledge gaps; incongruent with the expert role (Gulbrandsen et al., 2016).

Moreover, effective patient involvement and communication in the discharge process seem to be lacking. In a previous study, we reported that the patients had unstructured discharge processes not always tailored to meet their needs and that some did not have a discharge conversation at all (Rognan et al., 2021a). Lack of tailoring the discharge process to the patients’ needs has also been shown in previous studies where the patients did not feel ready for discharge, felt that their symptoms were not resolved, or there was a lack of information exchange from the HCPs (Flink & Ekstedt, 2017; Hestevik et al., 2019; Howard-Anderson et al., 2016; Rognan et al., 2021a). These findings align with Ubbink et al., who reported that different actors in the discharge process focus on different things and that doctors tend to focus on the medical conditions whereas nurses focus on the discharge procedures and the patient’s home situation (Ubbink et al., 2014). We suggest that more focus on communication and early patient involvement in the decision-making process is important for the patients’ self-management of their medications, and for their health outcomes.

All of the patients attempted resistance to the power relations at play in the day-to-day practices during their hospitalizations. Moreover, they were not granted an equal voice to the HCPs’ voices and were thus not able to interrupt the system’s objectification and subjectification of them. A part of this can be explained by the biomedical approach exercised by the HCPs which is shown by a preference to listen to other HCPs rather than the patient. Parts of the power structures are created in the differences in knowledge between patient and HCPs, communicative skills, and structural frameworks (such as staffing, number of patients, and less activity on the weekends).

This study indicates that discharge processes are complex and involve many relations, and one solution to all the presented issues is unlikely. We argue that a part of the issues stems from some HCPs being primarily trained in the biomedical discourse which discourages the use of SDM. Therefore, there is a need for HCPs to get more familiarized with SDM. One way to achieve this is to incorporate mentalization into the health care setting. Mentalization is the ability to understand people’s (but also one’s own) mental condition which lies behind the actions of others and oneself (i.e., feelings, needs, and wishes) (Gale et al., 2003). For HCPs, it is a tool that can help enhance communication, empathy, understanding, and identifying patients’ needs (Fogtmann, 2014). Meeting patients where they are will aid in tailoring consultations to the individual patient and meeting their needs.

## Strengths and limitations

A strength of this study is the richness of the data obtained by combining qualitative non-participant observations and semi-structured interviews (Simonÿ et al., 2018). For example, in the interviews, the patients could express their experiences more freely. The patients often had a positive attitude in their encounters with HCPs, however, in the interviews some criticism appeared. Observing patients in the hospital setting enables understanding of how the individual patients and HCPs interact, and to some degree it offsets the potential bias of the patient’s self-report (Thevelin et al., 2022).

The four patients included in this sub study did not differ from the other patients in the larger project by any visible characteristics, such as age, sex and education. We cannot exclude the possibility that these patients had a greater wish to be involved in decisions.

How we “entered the setting”, i.e., the internal medicines ward as observers, was critical for the validity of the results. A focus group study was performed to assess the effect of our observations on the HCPs, four weeks after the observations terminated (Svensberg et al., 2021). In addition, the semi-structured patient interviews included a question about how they had experienced being observed. Although the effect of the researcher in the social setting can never be controlled for, the results indicated that the observer effect in relation to medication communication seemed to be small and temporary (Svensberg et al., 2021). The medication communication appeared not to be strongly affected by the presence of the observers, and this strengthens the validity of the data. However, relations were built by eye contact or small talk in the corridor, confirmed by the patients’ description of how they perceived the observers as “my guardian” and “almost as my daughter”. One factor possibly contributing to success in observing the discharge process was that many HCPs reported a feeling of safety as they already “knew the observers” that is, were familiar with clinical pharmacists at the ward (Svensberg et al., 2021).

This study has some limitations. Narrative inquiries have been criticized for their perceived subjectivity and lack of generalizability (Beal, 2013; Connelly & Clandinin, 1990). However we had a small data set and never intended to generalize. Furthermore, observers filter what they register. In this study observers had an HCP background, hence the HCP perspective may have impacted the observations. How to adjudicate between the whole and the detail at each moment of the writing is a difficult task (Connelly & Clandinin, 1990). Narratives may be oversimplified for example when it comes to active versus passive, optimistic versus pessimistic, or powerless versus positive change and control over the future (Bochner & Riggs, 2014; Gale et al., 2003).

We choose not to use member check (also known as informant feedback or respondent validation) after the data was accessible in its restructured form. The reasons for this was Issues like recall bias and losing participants to follow up. The most important reason was however ethical, there is a risk of participant distress when viewing the spoken word in typed form, as well as “reliving” an potentially distressful period in one’s life.

The narratives presented in this article are not objective or complete but reflect the particular roles and positions of participants within the patient journey. Hence, the narratives should be viewed as suggestive, evocative, rather than final (Abma, 2002). The challenges shown in our research are similar to and add detail to research findings on SDM in the hospital setting from the patient perspective, and hence we assume our findings to also be relevant to other settings or contexts, that is not exclusively internal medicine wards in Norway.

We are aware that the word “patient” in many circumstances is not the most preferable to describe people participating in research (Chalmers, 1999). The people in the narratives we present were not just patients, they were self-managing individuals. However, we found the word “patient” appropriate in this study, to better distinguish between patient and HCP-participants.

In all research, the socio-cultural position of the researchers has an impact on the research process. The process of meaning construction (documentation of lives in files) is a relational and social process, and power inevitably plays a role in it (Abma, 2002). Analysts should remain vigilant and mindful of their obligations to the patient and the person-behind-the-patient and to the parts they themselves play in producing narratives (Bochner & Riggs, 2014). We addressed one aspect of this problem by involving a variety of different researchers because the patient’s voice should be the dominant one (Connelly & Clandinin, 1990). The researchers’ potential bias as well as measures to minimize this is described in the methods section.

## Conclusions

The four patients in this study were actively engaged in their treatments and had an information-seeking behaviour. However, even patients with a lot of knowledge and who challenged the HCPs’ expertise were not able to change the dynamics of the established power. Hence, the patients could not reach the SDM that they sought. To catalyse a change of the dominating biomedical discourse in health care towards more patient-centred care and SDM, the healthcare system and the individual HCP need to make more room for dialogue with the patients about their preferences. A part of this is also how health care systems are structured and scheduled, thus, it is important to empower patients and HCPs alike.
